# ID09, A Newly-Designed Tubulin Inhibitor, Regulating the Proliferation, Migration, EMT Process and Apoptosis of Oral Squamous Cell Carcinoma

**DOI:** 10.7150/ijbs.65824

**Published:** 2022-01-01

**Authors:** Qiushi Feng, Panpan Yang, He Wang, Congshan Li, Tomoka Hasegawa, Zhaopeng Liu, Minqi Li

**Affiliations:** 1 Department of Bone Metabolism, School and Hospital of Stomatology, Cheeloo College of Medicine, Shandong University & Shandong Key Laboratory of Oral Tissue Regeneration & Shandong Engineering Laboratory for Dental Materials and Oral Tissue Regeneration, Jinan, 250012, China; 2 Center of Osteoporosis and Bone Mineral Research, Shandong University, Jinan, China; 3 Department of Medicinal Chemistry, Key Laboratory of Chemical Biology (Ministry of Education), School of Pharmaceutical Sciences, Cheeloo College of Medicine, Shandong University, Jinan 250012, P. R. China; 4 Department of Developmental Biology of Hard Tissue, Graduate School of Dental Medicine, Hokkaido University, Sapporo 060-8586, Japan

**Keywords:** Tubulin Inhibitor ID09, Malignant Biological Behaviors, Apoptosis, Oral Squamous Cell Carcinoma, Mcl-1, Ras-Erk Pathway

## Abstract

Microtubules, a major target in oral squamous cell carcinoma (OSCC) chemotherapy, contribute to multiple malignant biological behaviors, including proliferation, migration, and epithelial-mesenchymal transition (EMT). Surpassing traditional tubulin inhibitors, ID09 emerges with brilliant solubility, photostability, and drug-sensitivity in multidrug-resistant cells. Its anti-tumor effects have been briefly verified in lung adenocarcinoma and hepatocellular carcinoma. However, whether OSCC is sensitive to ID09 and the potential mechanisms remain ambiguous, which are research purposes this study aimed to achieve. Various approaches were applied, including clone formation assay, flow cytometry, wound healing assay, Transwell assay, cell counting kit-8 assay, Western blot, qRT-PCR, and *in vivo* experiment. The experimental results revealed that ID09 not only contributed to cell cycle arrest, reduced migration, and reversed EMT, but accelerated mitochondria-initiated apoptosis. Remarkably, Western blot detected diminishment in expression of Mcl-1 due to the deactivation of Ras-Erk pathway, resulting in ID09-induced apoptosis, proliferation and migration suppression, which could be offset by Erk1/2 phosphorylation agonist Ro 67-7476. This study initially explored the essential role Mcl-1 played and the regulatory effect of Ras-Erk pathway in anti-cancer process triggered by tubulin inhibitor, broadening clinical horizon of tubulin inhibitors in oral squamous cell carcinoma chemotherapy application.

## Introduction

Ranked as the sixth most frequent malignancy worldwide, oral squamous cell carcinoma (OSCC) has the highest incidence among head and neck malignancies, whose 5-year survival rate is 63% 1. It accounts for 2.5% of cancer cases and 1.9% of all cancer-related deaths annually 2. Besides the morbidity and mortality following an upward trend 1, a rising trend of OSCC is observed among young patients all over the world 3. Clinical studies indicate that treatments in combination with chemotherapy are expected to exhaustively eradicate pre-invasive cancer cells and improve the prognosis of patients in an advanced stage to some extent, and the benefits of these treatments for the overall survival of patients with OSCC have been witnessed 1, 4. Unfortunately, more effective drugs need to be urgently designed considering the increasingly grim problem of anti-tumor drug resistance 4.

Composed of α-tubulin and β-tubulin heterodimer, microtubules are crucial components of the cellular cytoskeleton 5, which play an irreplaceable role in cell biological activities, ranging from cell morphology establishment and maintenance, spindle formation, chromosome segregation, intracellular cargo transportation to cell migration and so on 6, 7. In view of their pivotal role in eukaryotic cell survival, any interference to microtubules has an enormous possibility to incur relevant abnormality in cellular functions and even cell death 8, which makes them a major target in cancer therapy 6. Among researches on OSCC chemotherapy regimens, MDAs have rapidly emerged 9 by virtue of their exclusive features, including extensive anti-cancer spectrum, angiogenesis inhibition, and lethality to multi-drug resistance cell lines 10, 11. However, their way to the application has not been smooth due to the difficulties in development. A previous study has reported LL01, an indenoprazole compound combined with colchicine-binding site (CBS), as a potent MDA 12. Disappointingly, its low solubility and logP limit its clinical application 12, 13. Hence, ID09, a new optimized derivative of LL01 with excellent aqueous solubility and favorable logP value, was designed and synthesized 13. Its anti-cancer ability in restraining cell viability has been briefly explored earlier; however, the precise underlying molecular mechanism remains ambiguous. At the same time, its anti-tumor capacities manifested on other biological behaviors, such as proliferation, migration, EMT process and cell morphology, have not been explored yet.

As one of the ultimate destinations of cancer cells influenced by tubulin inhibitors 6, 8, apoptosis can be elucidated as programmed cell death aiming at homeostasis establishment and maintenance 14, 15. Apoptosis progress receives accurately adjustment from cytokines, and the core among these factors are caspases and B-cell lymphoma 2 (Bcl-2) family of proteins 14. As an anti-apoptotic member of the Bcl-2 subfamily, Mcl-1 was first discovered, extracted, and named from the human myeloid leukemia cell line ML-1 16. It exerts anti-apoptotic effects 17 via restraining both the release of cytochrome c from mitochondria and the origin of caspase cascade 18. However, the apoptosis variation via alteration of Mcl-1 is ignored by a majority of studies contraposing MDAs, not to mention the biological functions of Mcl-1 beyond apoptosis 17, 19, 20. The Ras-Erk pathway is construed as a quaternary-tier structure, involving Ras, Raf, Mek, and Erk 21. With the purpose of integrating a crucial approach to tackle a range of extracellular stimulus, the pathway is activated in the form of partial component phosphorylation 21, 22. The latest researches implied that the phosphorylation of Erk1/2 was seemingly associated with the increase in the Mcl-1 level 23, 24. However, the persuasive evidence for the role the Ras-Erk pathway played in fluctuations in the Mcl-1 level and the impact of MDA on the Ras-Erk pathway were still absent.

In the present study, ID09 was assessed as a tubulin inhibitor in OSCC therapy, focusing on malignant biological behaviors involving cell proliferation, migration, and EMT process. Considering the extensive adhibition in OSCC chemotherapy 1, 4 and its golden standard status in microtubule targeting drugs 25, paclitaxel (PTX) was selected as positive control in present study. Additionally, the underlying mechanisms of apoptosis mediated by Mcl-1 attenuation and Ras signaling pathway deactivation were also expounded, which is believed to broaden the understanding of the potential mechanisms of apoptosis induced by tubulin inhibitors.

## Materials and Methods

### Chemicals and reagents

ID09 was donated by Professor Zhaopeng Liu, School of Pharmacy, Shandong University. Erk phosphorylation agonist Ro 67-7476 and Mitochondrial protectant RU 360 were purchased from MedChemExpress (Shanghai, China). The antibodies against PCNA, MMP-2, MMP-9, β-Catenin, E-Cadherin, N-Cadherin, Vimentin, α-Tubulin, caspase-3, caspase-9, Cleaved PARP-1, Ras, Raf, p-Erk1/2, Erk1/2 cytochrome-c, Bak, Bad, Bax, Bcl-2, Bcl-xl and Mcl-1 were purchased from Abcam (Shanghai, China). Antibody against GAPDH was bought from Proteintech (Wuhan, Hubei, China).

### Cell lines and culture conditions

Human oral squamous carcinoma cell lines, SCC-15, Cal-27, SCC-25 and Tca8113 were obtained from Shanghai Cell Bank of Chinese Academy of Sciences (Shanghai, China) while human normal mucosa cells (HOK cell line) were purchased and identified from the Chinese Academy of Medical Sciences (Beijing, China). Human gingival fibroblasts (HGFs) were cultured from four patients undergoing maxillofacial surgery who were entirely free from clinical periodontal disease. The cells were cultured in DMEM containing 10% FBS (Gibco, Grand Island, NY, USA), and 1% penicillin-streptomycin at 37°C in humidified atmosphere of 5% CO_2_ and 95% air.

### Cell cycle flow-cytometry assay

The exponentially growing SCC-15 and Cal-27 cells were incubated in 6-well plates with a concentration of 3 × 10^5^/ well and allowed to adhere overnight. After incubation with different doses (0, 10, 15, 20 nM) of ID09 or paclitaxel (20 nM for positive control) for 24 hours, cells were harvested and fixed in ice-cold 70% ethanol overnight at 4°C, which was followed by centrifugation and rinse with ice-cold PBS. Subsequently, the cells were reacted with PI and RNase A (Elabscience, China) in dark at room temperature for 30 minutes. Finally, the cell cycle was acquired using a flow cytometer, and the data were analyzed with BD Accuri C6 plus software (Becton Dickinson, Franklin Lakes, NJ, USA).

### Western blot analysis

Total protein was isolated using RIPA lysis buffer, supplemented with protease inhibitor and phosphatase inhibitor (Beyotime, Beijing, China). The concentration of the protein was determined by BCA Protein Assay Kit (Beyotime, P0010, China). One-fourth volume of 5 x SDS loading buffer was mixed with each extract before heating at 95°C for 5 minutes. A total of 30 μg sample of corresponding groups was added and separated by 6-15% SDS-PAGE electrophoresis, followed by transfer to PVDF membrane. After being sealed with 5% BSA at room temperature for 1 hour, the membranes were incubated with correspondent primary antibodies, which were diluted according to instructions, and stayed overnight at 4°C. Subsequently the membranes were rinsed 3 times by TBST, followed by incubation with appropriate secondary antibody. After washing 3 times by TBST, the target protein bands were exposed using ECL detection system (SmartChemi 420, Beijing, China).

### Clone formation assay

The SCC-15 and Cal-27 cells were seeded in 6-well plates at a density of 500 cells/ well and incubated in standard culture condition overnight before being treated with different doses of ID09 (0, 10, 15, 20 nM) or PTX (20 nM for positive control) for 24 hours. After cultured for 14 days, the cell clones were cleaned with PBS, followed by fixation with 4% paraformaldehyde for 15 minutes and dyeing with 0.1% crystal violet for 15 minutes. Aiming at analyzing the data, clones that contained more than 50 cells were counted.

### Soft agar colony formation assay

Firstly, 0.7% and 1.2% agar were prepared and received autoclave sterilization before mixed with isometric cell culture medium containing double portion of FBS and penicillin-streptomycin. The 1.2% agar was added to 6-well plates and waited for solidification. In the meantime, the treated SCC-15 and Cal-27 cells were harvested and trace cell suspension containing 5 × 10^3^ cells was mixed with 0.7% agar and paved on 1.2% agar. The cells were allowed to culture for 14 day and culture medium was supplemented every three days. Finally, the cells were marked by MTT and representative pictures were photographed with a microscope (Olympus BX53, Tokyo, Japan).

### In-gel zymography assay

Total protein was isolated using RIPA lysis buffer without protease inhibitor and phosphatase inhibitors and separated by 8% sodium dodecyl sulfate polyacrylamide gel containing 1 mg/mL gelatin under 4°C environment, whose concentration was determined by BCA Protein Assay Kit (Beyotime, Beijing, China). Next, the gel received elution, renaturation, dye using Coomassie brilliant blue (Beyotime, Beijing, China) and rinse respectively before the inked ribbons were exposed using ECL detection system (SmartChemi 420, Beijing, China).

### Hoechst 33342-PI double staining

On the basis of the relationship between cell apoptosis and membrane permeability, Hoechst 33342-PI double staining kit (solarbio, Beijing, China) was utilized to detect cell apoptosis. The SCC-15 and Cal-27 cells received designed treatment, followed by staining via suitable amount of Hoechst 33342-PI mixed dye fluid for 30 minutes in dark. Eventually, a fluorescence microscope (Leica Dmi8, Wetzlar, Germany) was an effective method to record representative images.

### Mitochondrial membrane potential measurement

With the purpose of mitochondrial membrane potential (MMP) measurement, the SCC-15 and Cal-27 cells which have received corresponding treatment were rinsed by PBS directly *in situ* or after trypsinization. Simultaneously, JC-1 fluorescence probe working solution (Beyotime, Beijing, China) was also prepared according to instruction manual. Next, the OSCC cells was allowed to react with JC-1 probe for 20 minutes in dark and cleaned by staining buffer subsequently. Eventually, a fluorescence microscope (Leica Dmi8, Wetzlar, Germany) was utilized to directly photographed representative fluorescence pictures and a flow cytometry Accuri C6 plus (Becton Dickinson, Franklin Lakes, NJ, USA) was employed to detect the fluorescence quantitatively.

### Wound healing assay

The exponentially growing SCC-15 and Cal-27 cells were seeded into 6-well plates at a density of 3.5 × 10^5^/ well, and allowed to culture until 90% confluence. A perpendicular scratch was scratched on the surface of plate using a 200 μL pipette tip. After that, cells were incubated in 2% FBS-DMEM and treated with indicated doses of ID09 (0, 10, 15, 20 nM) or PTX (20 nM for positive control) for 24 hours. At 0, 24 and 48 hours after the scratch, the photographic images were acquired under a microscope (Olympus BX53, Tokyo, Japan) and analyzed by Image J software to calculate the healing percentage.

### Transwell assay

After incubation with indicated doses of ID09 (0, 10, 15, 20 nM) or PTX (20 nM for positive control), SCC-15 and Cal-27 cells were subsequently suspended in DMEM containing 3% FBS and seeded onto the upper chamber of the wells. Subsequently, 600 µL of DMEM containing 20% FBS was added to the lower chambers. After 24-hour invasion, cells through the membrane were fixed by 4% paraformaldehyde and stained by 0.1% crystal violet for 30 minutes. The cells were photographed with a light microscope (Olympus BX53, 100 Tokyo, Japan) at magnification and counted for analysis.

### RNA isolation and qRT-PCR

Total RNA was extracted from ID09 (0, 10, 15, 20 nM) treated cells or tumor tissue with the help of Trizol (TaKaRa, Tokyo, Japan), followed by reverse transcription using a SuperScript TM II reverse transcriptase kit (TaKaRa, Tokyo, Japan) according to the manufacturer's instructions. qRT-PCR labeled with SYBR Premix Ex Taq (TaKaRa Bio, Inc., Otsu, Japan) was executed in a Roche 480 LightCycler (Roche, Mannheim, Germany). The parameters required for denaturation, annealing, and extension were as follows: 95°C for 30 seconds, 45 cycles at 95°C for 5 seconds, and 60°C for 20 seconds. The gene levels of relative factors were presented by 2^-ΔΔCt^ and standardized to GAPDH.

The primers were synthesized as follows: GAPDH, Forward: 5'-CCTGCACCACCAACTGCTTA-3', Reverse: 5'-GGCCATCCACAGTCTTCTGAG-3'; MMP-2, Forward: 5'-CTCATCGCAGATGCCTGGAA-3', Reverse: 5'-TTCAGGTAATAGGCACCCCCCTTGAAGA-3'; MMP-9, Forward: 5'-CGCAGACATCGTCATCCAGT-3', Reverse: 5'-GGACCACAACTCGTCATCGT-3'; β-catenin, Forward: 5'-GGACCACAAGCAGAGTGCTGA-3', Reverse: 5'-TTCTGAACAAGACGTTGACTTGGA-3'; Vimentin, Forward: 5'-AACCTGGCCGAGGACATCA-3', Reverse: 5'-TCAAGGTCAAGACGTGCCAGA-3'; E-Cadherin, Forward: 5'-TGGAACAGGGACACTTCTGC-3', Reverse: 5'-CCCCGTGTGTTAGTTCTGCT-3'; N-Cadherin, Forward: 5'-CGAATGGATGAAAGACCCATCC-3', Reverse: 5'-GCCACTGCCTTCATAGTCAAACACT-3'; α-tubulin, Forward: 5'-CGACTCCTTCACCACCTTCTTCT-3', Reverse: 5'-CATCAATGACCGTAGGCTCCAGAT-3'; βI-tubulin, Forward: 5'-CGCAGAAGAGGAGGAGGATTT-3', Reverse: 5'-GGCAGTTGAGTAAGACGGCTAA-3'; βII-tubulin, Forward: 5'-GGCACGATGGATTCGGTTAGG-3', Reverse: 5'-ACACGAAATTGTCTGGTCTGAAG-3', βIII-tubulin, Forward: 5'-GAACCCGGAACCATGGACAG-3', Reverse: 5'-GACCCTTGGCCCAGTTGTTG-3', Bax, Forward: 5'-CCCGAGAGGTCTTTTTCCGAG-3', Reverse: 5'-CCAGCCCATGATGGTTCTGAT-3'; Bak, Forward: 5'-ATGGTCACCTTACCTCTGCAA-3', Reverse: 5'-TCATAGCGTCGGTTGATGTCG-3'; Bad, Forward: 5'-CAGTGACCTTCGCTCCACATC-3', Reverse: 5'-CCAAAGGAGACAGCACGGATC-3'; Bcl-2, Forward: 5'-TGGCCTTCTTTGAGTTCGGT-3', Reverse: 5'-GGGCCGTACAGTTCCACAA-3'; Bcl-xl, Forward: 5'-GACCATACTGAGGGACCAACTG-3', Reverse: 5'-GGGCCGTACAGTTCCACAA-3'; Mcl-1, Forward: 5'-ACAAAGCCAATGGGCAGGTC-3', Reverse: 5'-TTTGTTACGCCGTCGCTGAA-3'.

### Cell morphological investigation

The SCC-15 and Cal-27 cells were seeded at a density of 2 × 10^5^ cells/ well onto 6-well plates 24 hours before treatment with ID09 (0, 10, 15, 20 nM). After incubation for 24 hours, the cells were immediately photographed with a microscope (Olympus BX53, Tokyo, Japan). Fields were selected in the center of each well at approximately same location for photography.

### Cell viability assay

The SCC-15 and Cal-27 cells were planted in 96-well plates 24 hours before incubation at a density of 5 × 10^3^cells/ well, followed by treatment with ID09 or PTX at a wide range of concentrations (0, 1, 2, 4, 8, 16, 32, 64, 128 nM) for 24 hours or at 20 nM for different times (0, 12, 24, 36, 48, 60, 72 hours). CCK8 (MedChemExpress, China) was utilized to detect cell viability and the data of optical density at 450 nm was acquired from a Bio-Rad Microplate Reader (Model 680, Bio-Rad, USA).

### Apoptosis flow-Cytometry assay

The SCC-15 and Cal-27 cells was planted in 6-well plates at 2 × 10^5^ cells/ well. After designative ID09 or PTX treatment for 24 hours, the cells were harvested and washed twice with ice-cold PBS, followed by suspension in 100 µL 1 × binding buffer. After incubated by 5 µL Annexin V-FITC and 5 µL PI (BD Biosciences, China) at room temperature in dark for 10 minutes, cell suspension was mixed with another 400 µL of 1 × binding buffer. Finally, cell apoptosis was measured by flow cytometry using Accuri C6 plus (Becton Dickinson, Franklin Lakes, NJ, USA) within an hour.

### Animal model and tissue preparation

All animal experiments were executed in accordance with the Guidelines for Animal Experimentation of Shandong University. The male BALB/c nude mice (6-weeks old) were purchased from Jinan Pengyue Experimental Animal Breeding Co., Ltd. (Shandong, China). The SCC-15 xenograft model was established by injecting 2 × 10^6^ SCC-15 cells suspended in 200 μL PBS at the right armpit of mice. When the tumor volume reached 50 mm^3^, nude mice were randomly divided into two groups: vehicle group (distilled water, iv, n = 4) and ID09 group (10 mg/kg, iv, n = 4). ID09 was dissolved in distilled water to prepare a 2.0 mg/mL solution for intravenous injection every two days for 21 consecutive days, as what has been reported before 13. Tumor volumes (volume = length × width^2^ /2) were measured every 3 days during drug treatment. When the administration ended, the mice were sacrificed, and the tumors were removed and measured. The protein expression levels of GAPDH, PCNA, Mcl-1, MMP-2, MMP-9, Vimentin, E-Cadherin, N-Cadherin, β-catenin, cleaved caspase-3, cleaved caspase-9 and cleaved PARP-1 in the tumor tissues were detected by Western blot. All animal experimental procedures were approved by NO.20210511.

### Histological examination

After dewaxing and hydration, the sections were soaked in hematoxylin dye for 15 minutes, followed by rinse with distilled water. Subsequently, the sections were stained with eosin dye for 7 minutes and rinsed with distilled water. Then the slices were dehydrated with an ascending alcohol concentration gradient, soaked in xylene solution twice, and then mounted with neutral gum. The representative pictures of each group were obtained using an optical microscope (Olympus BX-53, Tokyo, Japan).

### Immunohistochemical examination

After incubation with 0.3% hydrogen peroxide for 30 minutes at room temperature, the dewaxed paraffin sections were pre-treated with 1% BSA-PBS for 20 minutes at room temperature. Next, the sections were treated with the primary antibody (anti-PCNA, 1: 100) among BSA-PBS environment at room temperature. Two hours later, the sections were cleaned by PBS and treated with the related secondary antibody (1: 200) for 1 hour at room temperature. Diaminobenzidine (Sigma-Aldrich, MO, USA) was used as the substrate for immune complexes to visualize. Sections were feebly counterstained with methyl green for evalution and images were acquired by an optical microscope (Olympus BX-53, Tokyo, Japan) and digital images were taken.

### TdT-mediated dUTP nick-end labeling

Aiming at analyze cell apoptosis in tissue, a one-step TUNEL assay kit (Elabscience, China) was employed. The paraffin sections were dewaxing by xylene, soaked in gradient ethyl alcohol, rinsed by PBS and pre-treated by protease K fluid respectively before labeling. Next, TdT equilibration buffer and labeling fluid were prepared and applied to mark dUTP nick-end in dark. After rinsed by PBS, the sections were sent to observed and photographed under a fluorescence microscope (Leica Dmi8, Wetzlar, Germany) after cell nucleus redyeing by DAPI.

### Statistical analysis

All the experiments above were repeated at least three times independently and expressed as means ± standard deviations. Image J and Graphpad Prism 7 were utilized to analyze the statistics obtained. T-test were performed for comparison between two groups and one-way analysis of variance (ANOVA) for differences across more than two groups. A P < 0.05 was regarded as statistical significance.

## Results

### ID09 prevented proliferation of SCC-15 and Cal-27 cells, resulting in cell cycle arrest in the S and G_2_/M phases

**Figure 1:**
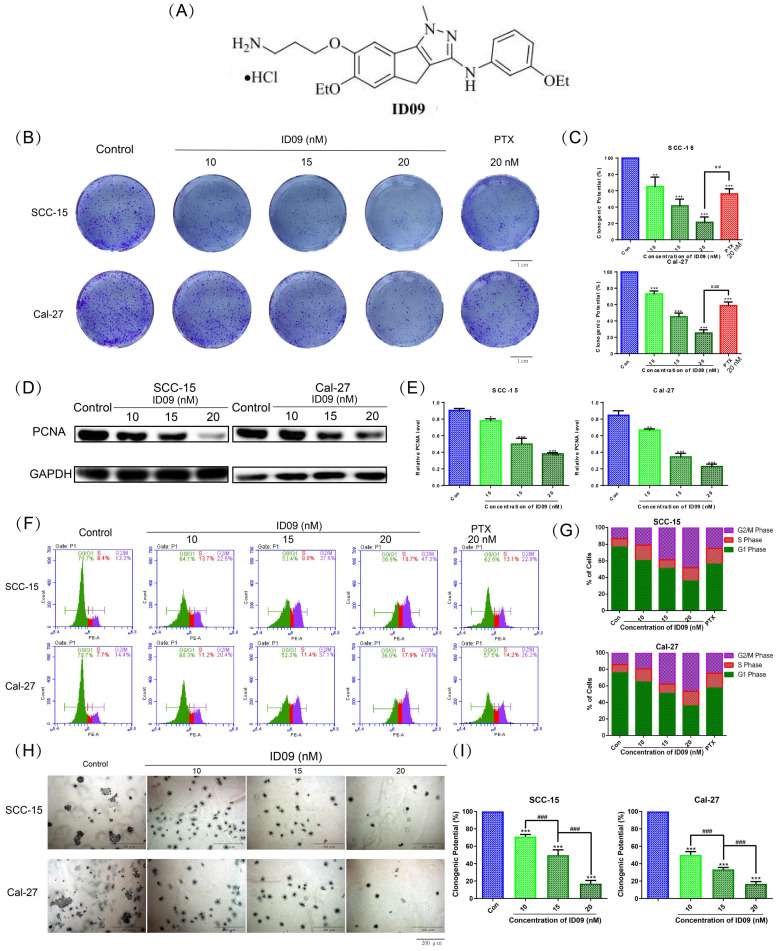
Proliferation of SCC-15 and Cal-27 cells was suppressed by ID09. (A) Chemical structure of ID09. (B and C) Clone formation assay was performed after treating cells with different doses of ID09 (0, 10, 15, and 20 nM) or PTX (20 nM, positive control) for 24 hours. (D and E) Western blot detected PCNA expression level in SCC-15 and Cal-27 cells with indicated doses of ID09 (0, 10, 15, and 20 nM). (F and G) Flow cytometry was used to test the cell cycle after the cells were treated with ID09 (0, 10, 15, and 20 nM) or PTX (20 nM, positive control), revealing that ID09 could arrest cell cycle in the S and G_2_/M phases. (H and I) Soft agar colony formation assay was effective in cell anchorage-independent growth ability measurement. The columns represent the means, and error bars represent standard deviations (NS, non-significant difference; */#P < 0.05; **/##P < 0.01; ***/### P < 0.001 versus the indicated group).

The chemical structure of ID09 is shown as Figure 1A. Microtubule depolymerization can obstruct cell proliferation 6. Conforming to this theory, the clone formation assay unveiled that ID09 distinctly diminished the valid clones of SCC-15 and Cal-27 cells in a dose-dependent manner, compared with negative control and positive control groups (Fig. 1B and 1C). The descending expression level of PCNA due to ID09 treatment in a dose-dependent manner was detected by Western blot (Fig. 1D and 1E). Cell cycle arrest is a typical manifestation of cell proliferation reduction 26, 27. Therefore, flow cytometry was applied to detect the cell cycle distribution among OSCC cells treated with ID09 or PTX. Contrary to the decrease in the G_0_/G_1_ phase cell population, the proportion of S phase and G_2_/M phase cells obviously increased as the dosage of ID09 increased, whose effect excelled that of PTX in the same dosage (Fig. 1F and 1G). In order to examine cell anchorage-independent growth ability in OSCC cells treated with gradient dose of ID09, soft agar colony formation assay was performed. It is obvious that both volume and count of SCC-15 and Cal-27 cell clone were impaired by ID09 incubation in a dose-dependent manner according to representative pictures and cartogram exhibited in Figure 1H and 1I.

### ID09 inhibited the migration and invasion of SCC-15 and Cal-27 cells

**Figure 2:**
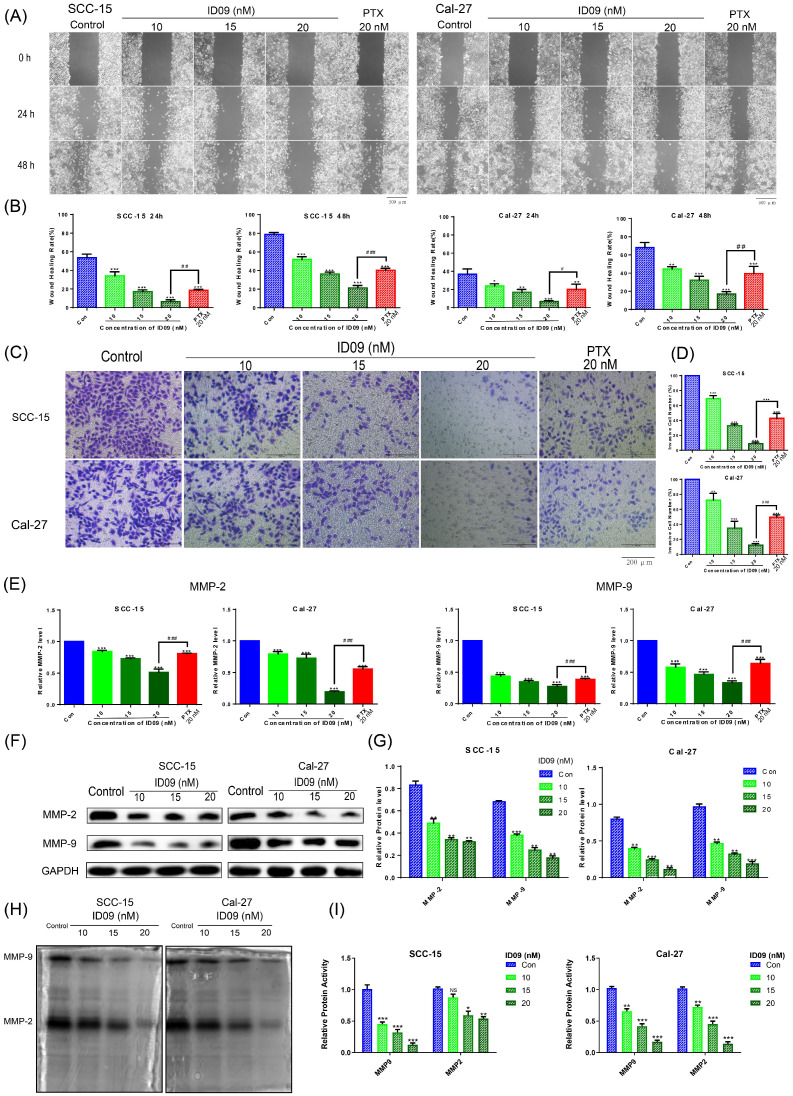
Effects of ID09 on the migration and invasion of SCC-15 and Cal-27 cells. (A and B) Wound healing rates in SCC-15 and Cal-27 cells after 24 and 48 hours were reduced by ID09 or PTX (positive control) in a dose-dependent manner. (C and D) Representative pictures of the Transwell assay performed in SCC-15 and Cal-27 cells treated with ID09 (0, 10, 15, and 20 nM) and PTX (20 nM, positive control) were exhibited, and the tendency of invasive cells was analyzed. (E) qRT-PCR was employed to measure the variation trend in mRNA expression levels of MMP-2 and MMP-9 in SCC-15 and Cal-27 cells treated with ID09 (0, 10, 15, and 20 nM) and PTX (20 nM, positive control). (F and G) Western blot was applied to measure the protein level of MMP-2 and MMP-9 in SCC-15 and Cal-27 cells treated with ID09 (0, 10, 15, and 20 nM). (H and I) In-gel zymography assay was selected to detect the activities of MMP2 and MMP9 in SCC-15 and Cal-27 cells. The columns represent the means, and error bars represent standard deviations (NS, non-significant difference; */#P < 0.05; **/##P < 0.01; ***/### P < 0.001 versus the indicated group).

A comparison of negative and ID09 groups in wound healing assay showed that the healing rates of SCC-15 and Cal-27 cells were distinctly restrained by ID09 incubation, which were more obvious than those in the positive control group (Fig. 2A and 2B). Meanwhile, representative pictures and cell count from Transwell assay suggested that ID09 diminished more cells through the membrane compared with PTX (Fig. 2C and 2D). Subsequently, qRT-PCR was utilized to analyze MMP-2 and MMP-9 mRNA levels, showing that MMP-2 and MMP-9 mRNA levels decreased after incubating with ID09, compared with those in the negative and positive control groups (Fig. 2E). Meanwhile, Western blot results also verify that ID09 incubation results in MMP-2 and MMP-9 expression diminishment from protein level (Fig. 2F and 2G). Furthermore, in-gel zymography serves as a more sensitive methodology to detect the activities of MMP-2 and MMP-9, whose results indicate that ID09 incubation significantly reduces the enzymatic activity of MMP-2 and MMP-9 (Fig. 2H and 2I).

### ID09 altered the morphology of SCC-15 and Cal-27 cells and reversed the epithelial-mesenchymal transition process via regulating microtubules

**Figure 3:**
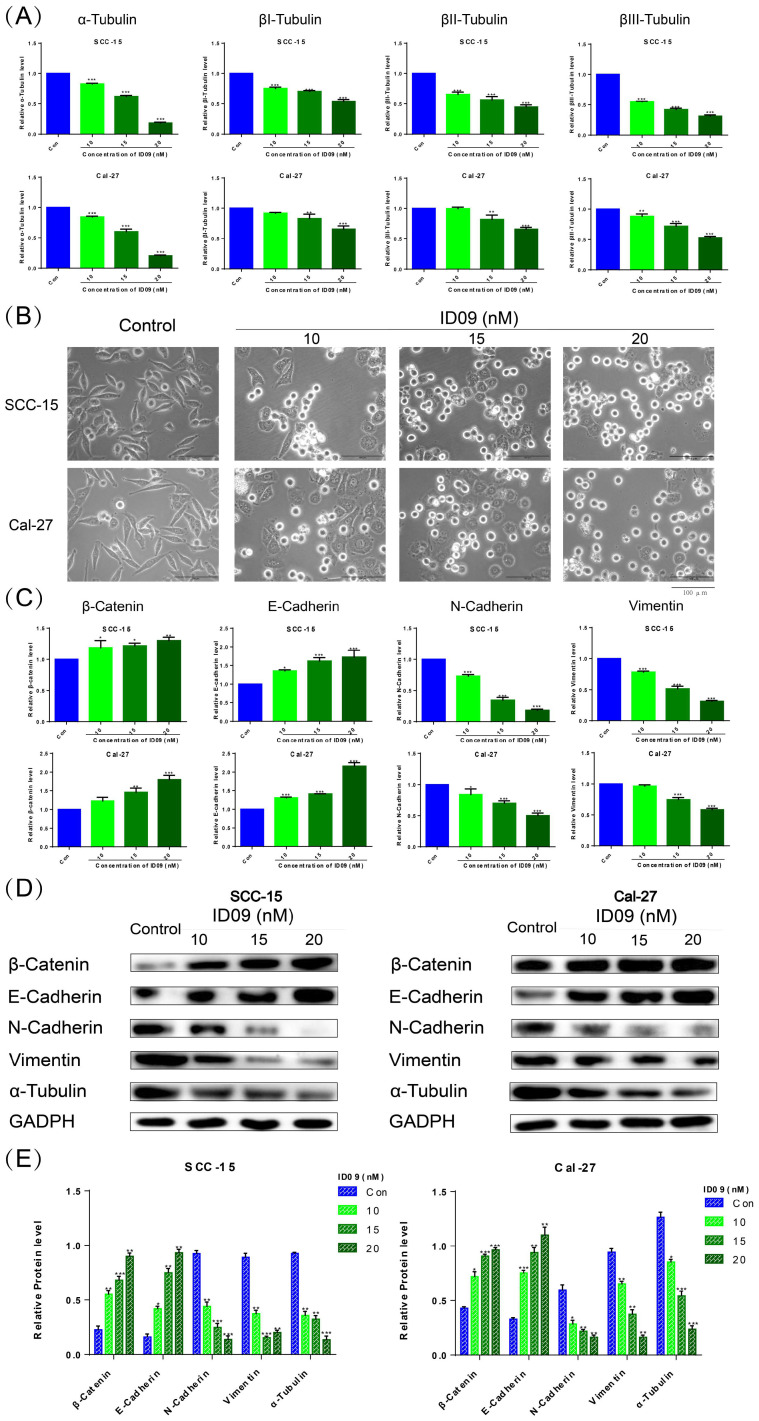
ID09 influenced SCC-15 and Cal-27 cells morphology and EMT process through regulation of microtubules. (A) qRT-PCR was applied to detect the mRNA level variations of four kinds of tubulins, and (C) factors associated with epithelial-mesenchymal transformation were tested as well. (B) The cellular morphology changes induced by ID09 were photographed by microscope. (D and E) Western blot was used as a method to gauge β-catenin, E-Cadherin, N-Cadherin Vimentin, and α-tubulin expression levels. The columns represent the means and error bars represent standard deviations. (NS, non-significant difference; */#P < 0.05; **/##P < 0.01; ***/### P < 0.001 versus the indicated group).

qRT-PCR was utilized to detect mRNA levels of α, βI, βII, and βIII tubulins polymerizing into microtubules (Fig. 3A). The expressions of four kinds of tubulins were attenuated to different degrees compared with the negative control group, especially α-tubulin. And the ID09-mediated diminishment in α-tubulin expression was verified by Western blot (Fig. 3D and 3E). The cell morphology was photographed and representative images are exhibited as Figure 3B. Interestingly, the logarithmic proliferating cells transformed from the mesenchymal state with a spindle shape to the epithelial state with a polygonal shape attributed to incubation with low-dose ID09 (10 nM). With the augmentation of concentration (15 nM and 20 nM), part of cells became round due to tension after cytoskeleton destruction, eventually leading to death. Meanwhile, β-catenin, E-Cadherin, N-Cadherin and vimentin were assessed by qRT-PCR as biomarkers on behalf of the EMT process. As results exhibited, the mRNA expression levels of β-catenin and E-Cadherin increased while the expression of N-Cadherin and vimentin decreased due to ID09 incubation (Fig. 3C), indicating the reversal of the EMT process. In line with qRT-PCR results, Western blot shows that the protein expression levels of β-catenin and E-Cadherin up-regulated while the expression of N-Cadherin and vimentin down-regulated due to ID09 incubation, which is a symbol of EMT process reversion (Fig. 3D and 3E).

### ID09 stimulated SCC-15 and Cal-27 cell apoptosis mediated by the caspase cascade via the mitochondrial mechanism

**Figure 4:**
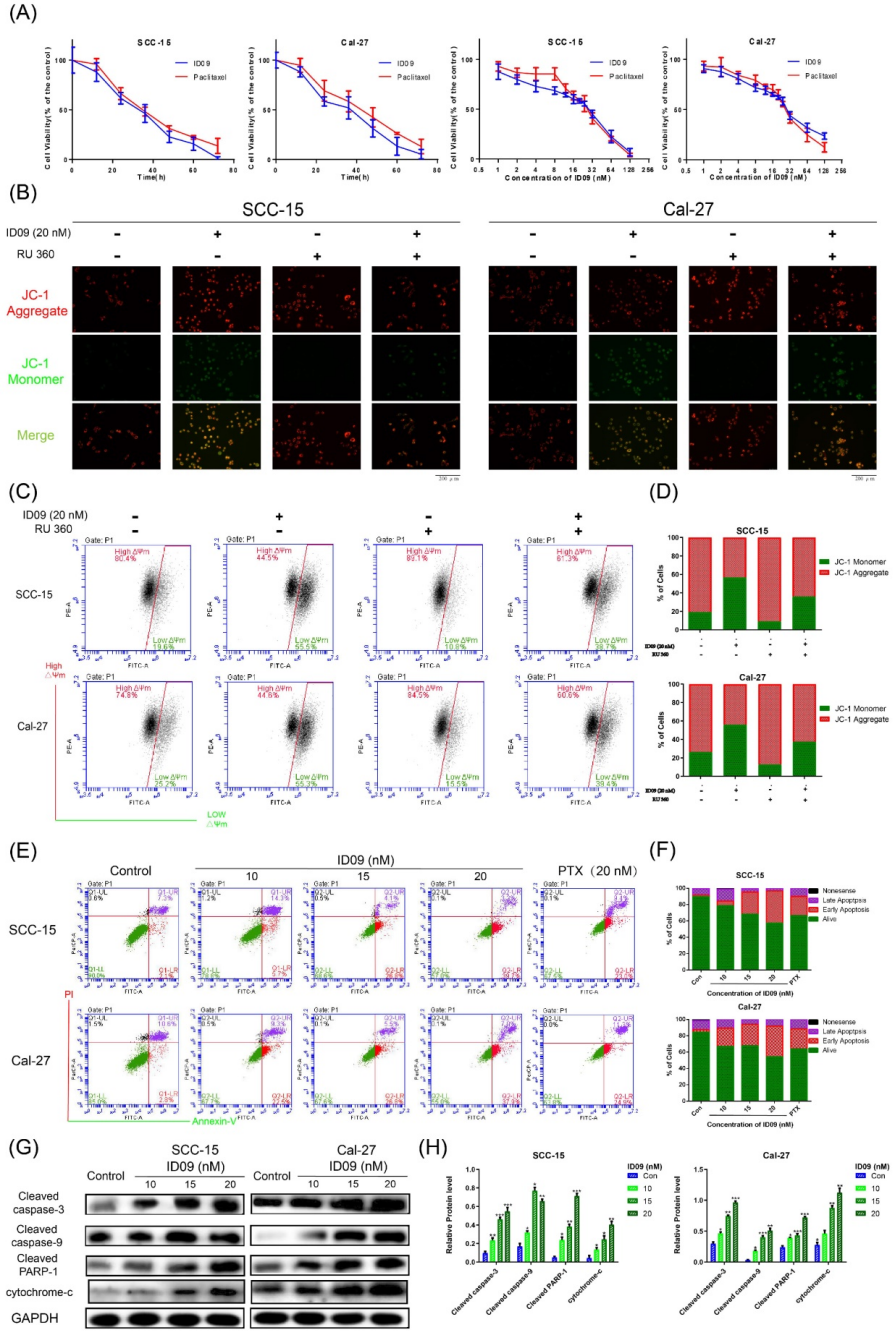
Apoptosis via mitochondrial mechanism occured in SCC-15 and Cal-27 cells as a result of ID09 incubation. (A) CCK-8 Assay was used for detection to viability of SCC-15 and Cal-27 cells treated with different doses (0, 1, 2, 4, 8, 16, 32, 64, 128 nM) of ID09 for 24 hours or with 20 nM ID09 for different durations (0, 12, 24, 48, 60, 72 hours), PTX under same condition was used as a positive control. (B) Fluorescence pictures of OSCC cells marked by JC-1 probe were exhibited to detect mitochondrial membrane potential variation visually. (C and D) Mitochondrial membrane potential of SCC-15 and Cal-27 cells treated with ID09 were quantificationally measured via flow cytometry. (E and F) Flow cytometry was applied to test apoptosis degree of SCC-15 and Cal-27 cells incubated with ID09 or PTX (20 nM for positive control). (G and H) Western blot was utilized to measure the levels of apoptosis associated proteins extracted from cells incubated with ID09 (0 10 15 20 nM) for 24 hours, and the data were analyzed by Image J. The columns represent the means and error bars represent standard deviations. (NS, non-significant difference; */#P < 0.05; **/##P < 0.01; ***/### P < 0.001 versus the indicated group).

Microtubule depolymerization is very likely to cause cell apoptosis. The CCK8 assay was performed at a wide range of concentrations or times to test cell viability. As shown in Figure 4A and Supplementary data 1A, the viabilities of four kinds of OSCC cell lines, consisting of SCC-15, Cal-27, Tca8113 and SCC-25 were apparently reduced by ID09 in a dose and time dependent manner. The effect of ID09 were slightly superior to paclitaxel under the same conditions, especially in low concentration region. Meanwhile, IC_50_ of ID09 in each OSCC cell line was calculated to guide further research (Supplementary data 1B and 1C). However, the cell viability attenuation in normal non-cancerous cell lines HOK and HGF were partly weaker than in OSCC cell line (Supplementary data 1D). On the other hand, JC-1 fluorescent images of SCC-15 and Cal-27 are displayed to illustrate that ID09 remarkably destroyed the integrity of the mitochondrial membrane, which can be saved by mitochondrial protectant RU 360 (Fig. 4B). In a similar way, flow cytometry unveiled that mitochondrial membrane potential of SCC-15 and Cal-27 cells were descended due to ID09 treatment and this trend was reversed by RU 360 (Fig 4C and 4D). Subsequently, Annexin V/PI double staining was employed to identify the cell apoptosis in SCC-15 and Cal-27 cells (Fig. 4E and 4F). Compared with the negative group, the proportion of cells in early apoptosis was effectively increased by ID09 in a dose-dependent manner, whose effect preceded that of PTX at the same dose. In addition, Western blot showed that the expression of cleaved caspase-3, cleaved caspase-9, cleaved PARP-1 and cytochrome-c were also enhanced by ID09 with increase in concentration (Fig. 4G and 4H).

### ID09 down regulated the Mcl-1 level in SCC-15 and Cal-27 cells by inhibiting the Ras-Erk signaling pathway

**Figure 5:**
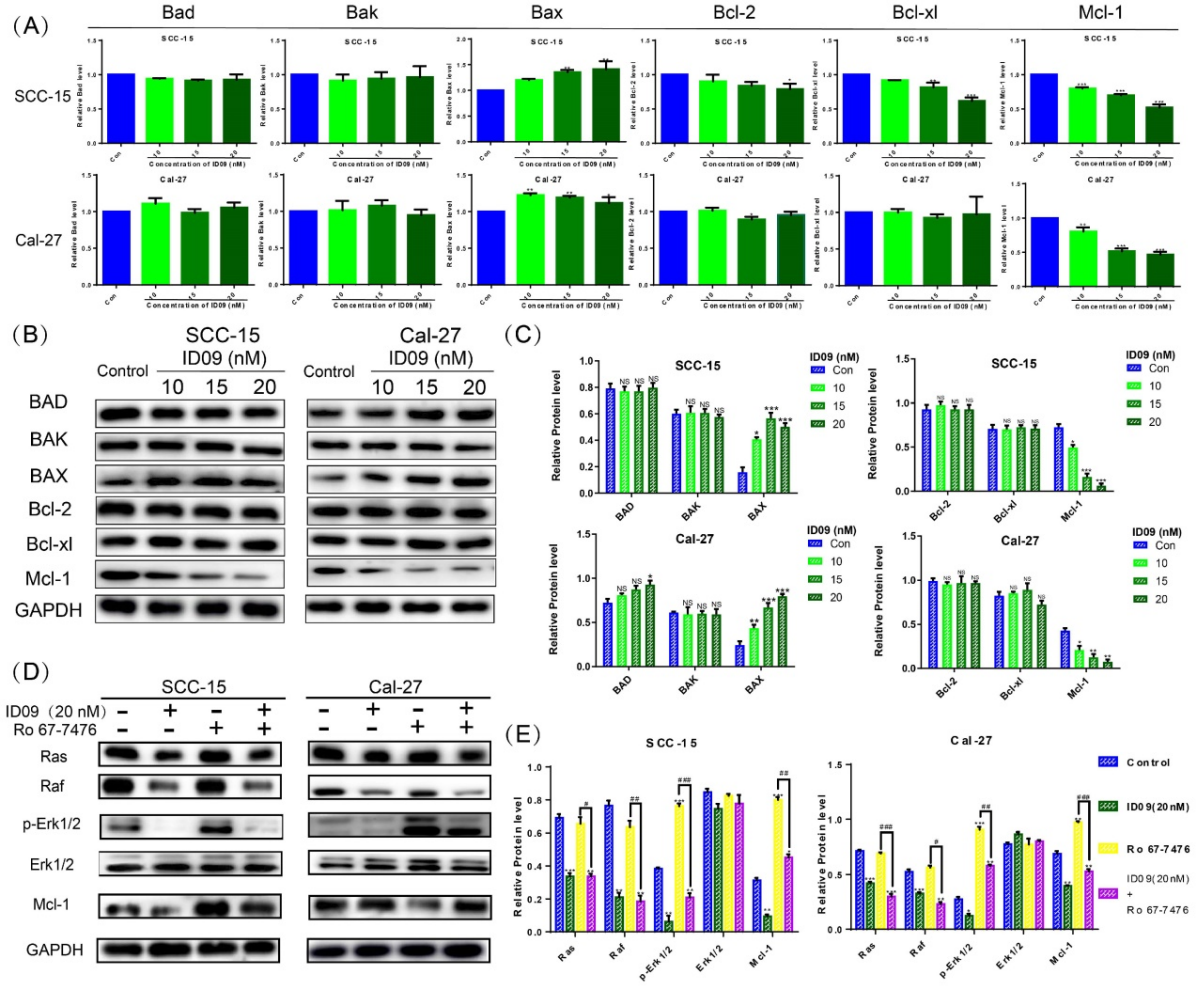
The effects of ID09 on Ras-Erk signaling pathway and Mcl-1. (A) qRT-PCR was utilized to detect the mRNA level variations of Bcl family factors. (B and C) Western blot was applied to verify the influence of ID09 treatment on Bcl family factors from protein level and (D and E) gauge the activation degree of Ras-Erk signaling pathway in treated SCC-15 and Cal-27 cells. The columns represent the means and error bars represent standard deviations. (NS, non-significant difference; */#P < 0.05; **/##P < 0.01; ***/### P < 0.001 versus the indicated group).

The mRNA levels of Bcl family proteins, including Bad, Bak, Bax, Bcl-xl, Bcl-2 and Mcl-1, were evaluated by qRT-PCR. Although major factors showed no significant change, the expression of Mcl-1 was significantly attenuated, hence the expression of Bax was slightly augmented by ID09 (Fig. 5A). Simultaneously, the ID09-mediated variation of Bad, Bak, Bax, Bcl-xl, Bcl-2 and Mcl-1 were verified via Western blot, reappearing the decrease in Mcl-1 and increase in Bax (Fig. 5B and 5C). Assessed by Western blot, the activation of the Ras-Erk signaling pathway, represented by Ras, Raf, and p-Erk1/2, was down regulated by ID09 treatment. However, the phosphorylation of Erk1/2 and expression level of Mcl-1 were relieved by Erk1/2 phosphorylation agonist Ro 67-7476 (1 μM for 5 min) 28 in the ID09 + Ro 67-7476 group compared with the ID09 group (Fig. 5D and 5E).

### Ras-Erk signaling pathway reactivation offsets the ID09-related proliferation inhibition, migration restraining and invasion suppression in SCC-15 and Cal-27 cells

**Figure 6:**
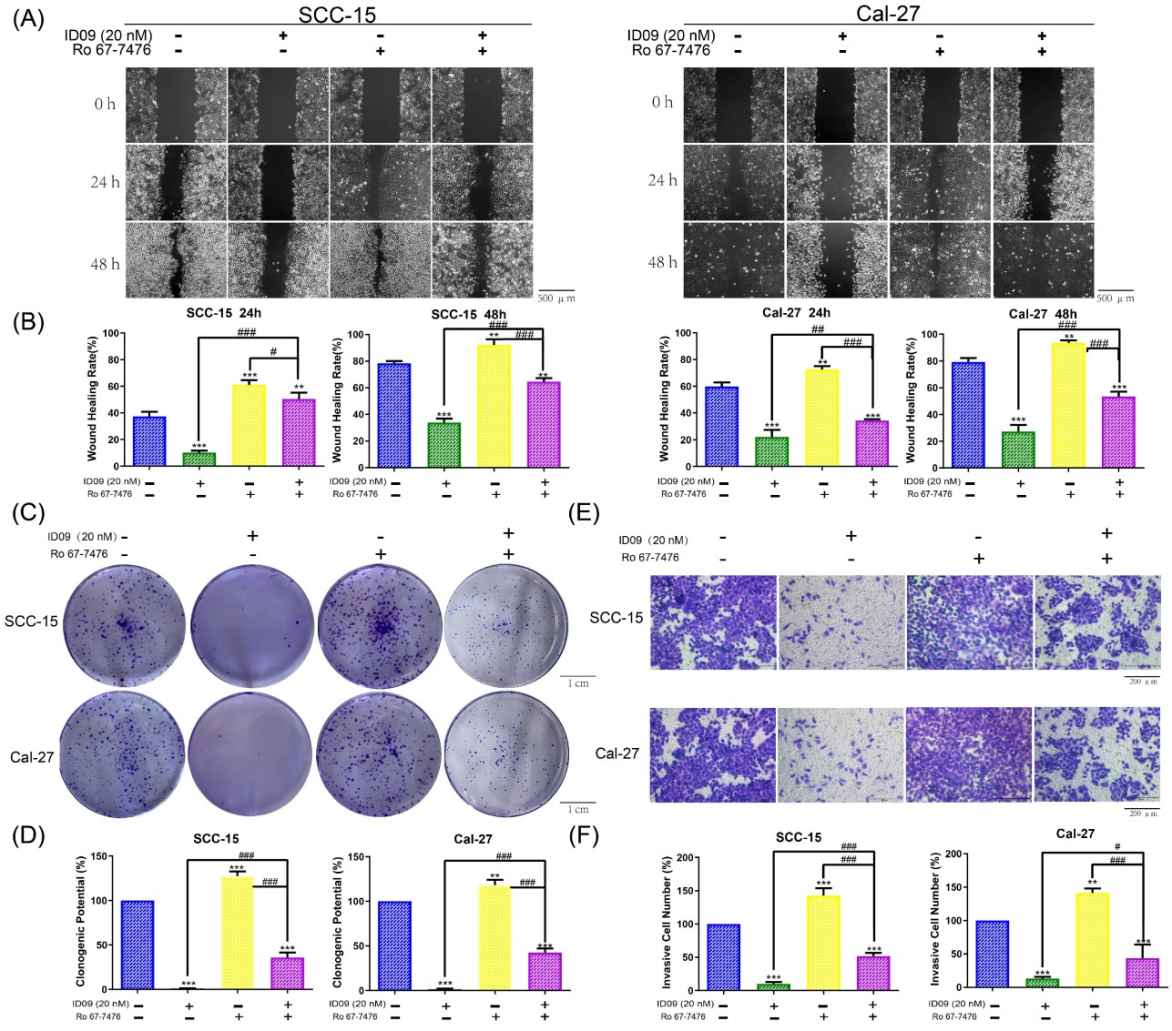
The proliferation inhibition, migration restraining and invasion suppression of SCC-15 and Cal-27 cells from ID09 incubation could be remedied by Ras pathway agonist Ro 67-7476. (A and B) Wound healing rates in SCC-15 and Cal-27 cells after 24 and 48 hours were reduced by ID09 but saved by Ro 67-7476. (C and D) The pictures of clone formation assay performed in OSCC cells incubated with ID09 and/or Ro67-7476 were displayed above. (E and F) Transwell assay was selected to measure the influence of ID09 and Ro 67-7476 on OSCC cells invasive ability. The columns represent the means and error bars represent standard deviations. (NS, non-significant difference; */#P < 0.05; **/##P < 0.01; ***/### P < 0.001 versus the indicated group).

In order to explore the regulatory effect of Ras-Erk pathway in the anti-cancer process triggered by the tubulin inhibitor, loss-of-function and gain-of-function assays were performed. Notably, assessed by wound healing assay, wound healing rates of SCC-15 and Cal-27 cells impaired by ID09 were partly repaired after Ro 67-7476 incubation, which could promote wound healing solely (Fig. 6A and 6B). On the other hand, the images displayed in Figure 6C and 6D declared that Ro 67-7476 was capable of accelerating cell clone formation and reversing adverse effect from ID09 to valid clone formation. Meanwhile, the invasive cell count from Transwell assay demonstrated that Ro 67-7476 possessed antagonism against ID09 effect that restrained SCC-15 and Cal-27 cells through the membrane (Fig. 6E and 6F).

### Ras-Erk signaling pathway reactivation attenuated the cell apoptosis of SCC-15 and Cal-27 cells caused by ID09 treatment

**Figure 7:**
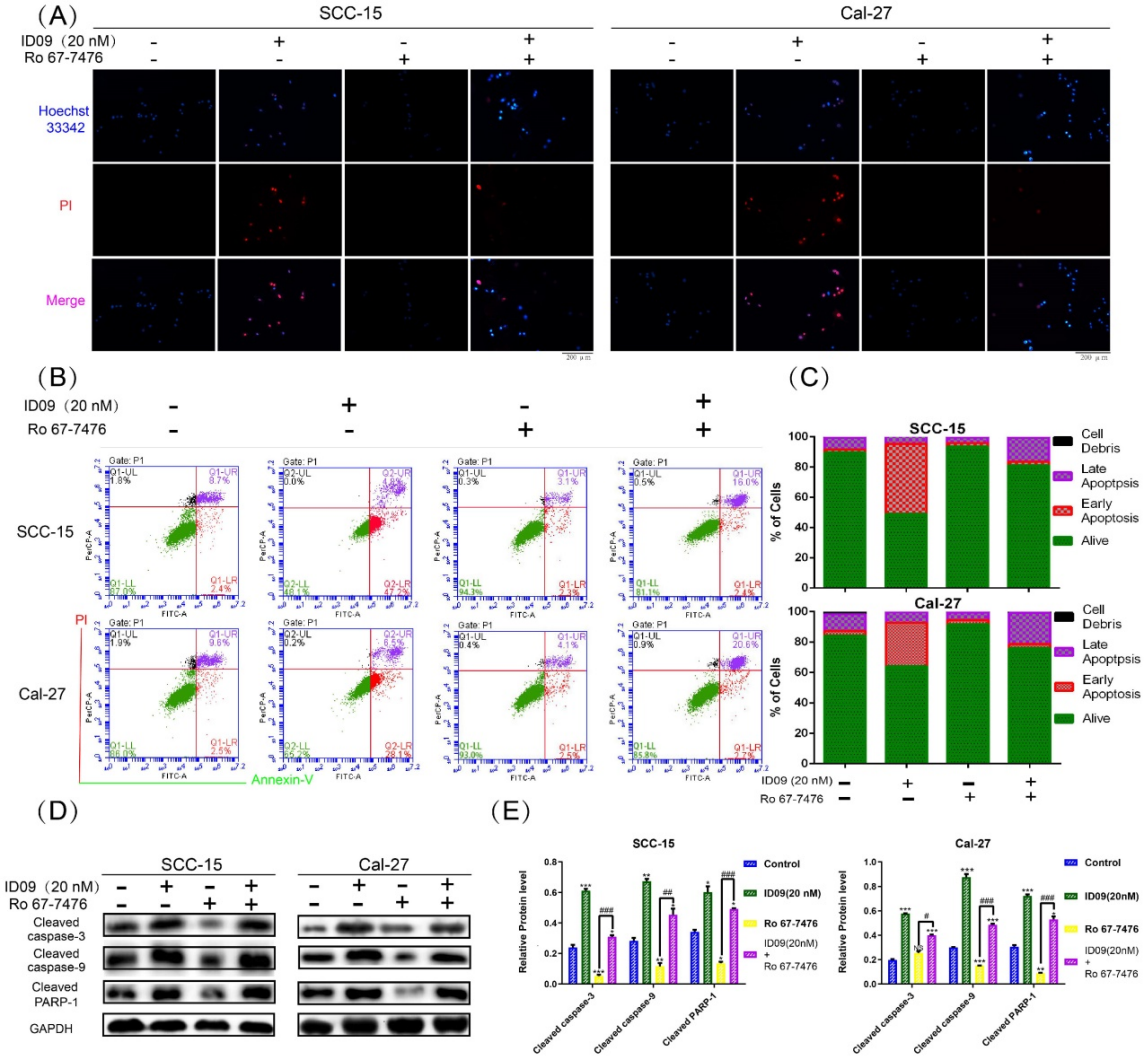
Ras-Erk pathway agonist Ro 67-7476 is able to salvage ID09-mediated apoptosis in SCC-15 and Cal-27 cells. (A) Survival, apoptosis and necrosis in incubated OSCC cells were demonstrated visually via Hoechst 33342-PI double stain and (B and C) calculated mathematically by flow cytometry. (D and E) Western blot was utilized to analyze the variation of mitochondria apoptosis related factors inSCC-15 and Cal-27 cells. The columns represent the means and error bars represent standard deviations. (NS, non-significant difference; */#P < 0.05; **/##P < 0.01; ***/### P < 0.001 versus the indicated group).

Hoechst 33342-PI double staining was an efficient method to identify cell survive, apoptosis and necrosis. As exhibited in Figure 7A, apoptotic cells increased significantly after ID09 incubation, which can be marked by high light blue-fluorescence from Hoechst 33342 and red-fluorescence from PI. However, this phenomenon had been offset after Ras-Erk signaling pathway reactivation via Ro 67-7476 treatment. Quantificationally, flow cytometry was utilized to analyze cell apoptosis of SCC-15 and Cal-27 cells. As prolepsis, in comparison with control group, the apoptosis ratio in SCC-15 and Cal-27 cells was dramatically augmented by ID09 but mildly diminished by Ro 67-7476. When applied simultaneously, Ro 67-7476 rescued OSCC cells from apoptosis to a certain extent in the ID09 + Ro 67-7476 group compared with ID09 group (Fig. 7B and 7C). The protein levels of caspase cascade, including cleaved caspase-9, cleaved caspase-3 and cleaved-PARP1, were weakened in the ID09 group, which could be saved by Ro 67-7476 compared with that in the ID09 + Ro 67-7476 group (Fig. 7D and 7E).

### Anti-tumor effects of ID09 in tumor xenograft

**Figure 8:**
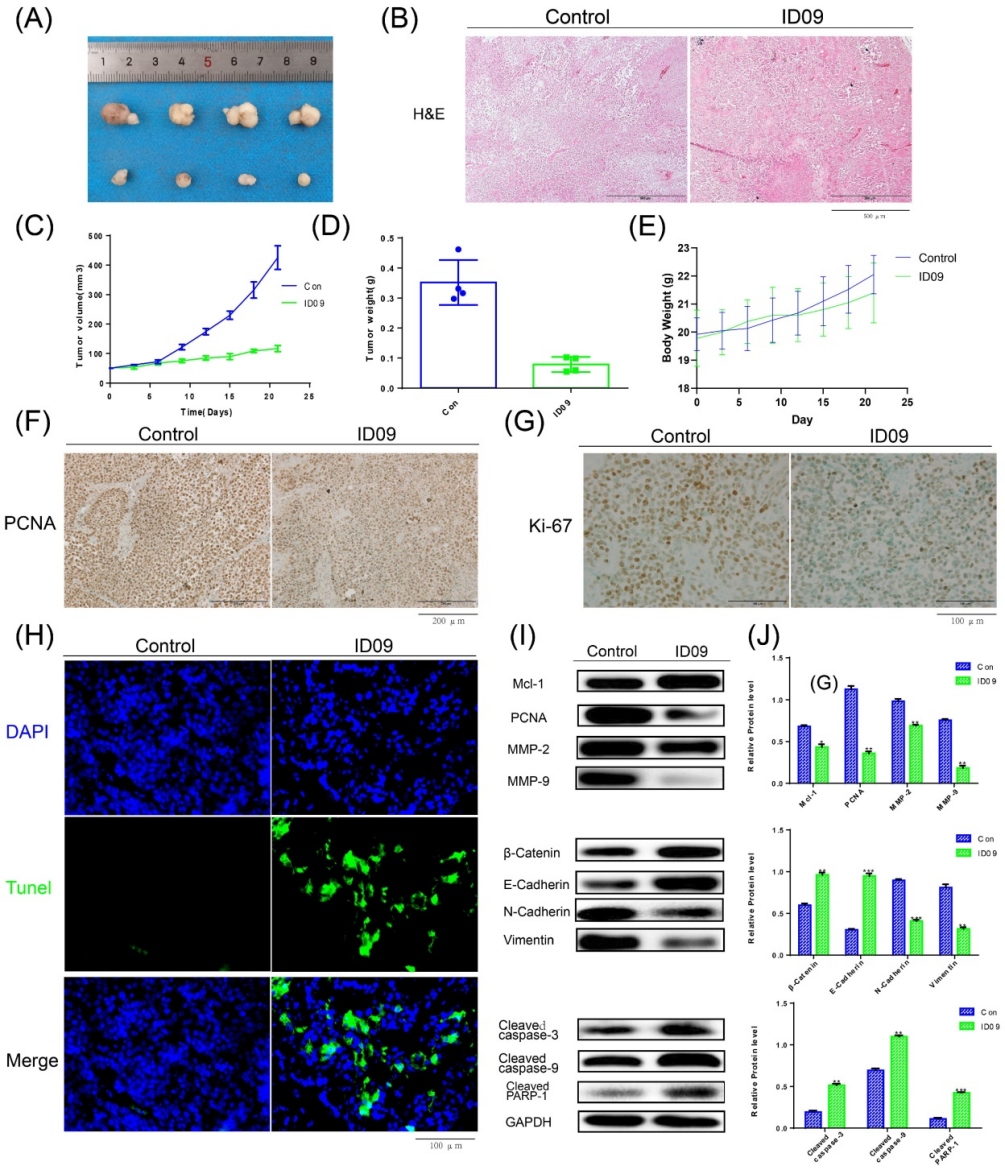
Anti-tumor efficiency of ID09 on SCC-15 xenografts. (A) Picture of xenograft tumor was exhibited as follow. (B) HE staining was implemented to perform histological examination. (B, C and D) Average tumor volumes, tumor weights and body weights variation of each mice group were measured. (F and G) The expression of PCNA and Ki-67 in tumor tissue after ID09 treatment was visualized by IHC staining. (H) Cell apoptosis in tissue incubated by ID09 was detected by TUNEL staining. (I and J) Protein extracted from tumor tissue were determined by Western blot. The columns represent the means and error bars represent standard deviations. (NS, non-significant difference; */#P < 0.05; **/##P < 0.01; ***/### P < 0.001 versus the indicated group).

The images of xenograft tumors demonstrated that ID09 intravenous injection suppressed SCC-15 cell-derived xenograft tumor growth in terms of size, volume, and weight (Fig. 8A, 8C and 8D), while led to no significant effect on the body weight of nude mice (Fig. 8E). The change in morphology was evaluated by HE staining, which showed the abnormality of cell shape (Fig. 8B); the expressions of PCNA and Ki-67 detected by IHC staining were obviously diminished (Fig. 8F and 8G). TUNEL staining demonstrated that compared with the control group, the tumor cells in the tissue were significantly apoptotic after ID09 injection (Fig. 8H). Western blot was applied to measure the mitigation of Mcl-1, PCNA, MMP-2, MMP-9, biomarkers of EMT process and the activation of caspase cascade, reflecting the influence of ID09 to tumor malignant biological behaviors *in vivo* (Fig. 8I and 8J).

## Discussion

OSCC is known as the most common malignancy of the head and neck, whose morbidity rocket rapidly, especially in young and middle-aged people 1, 3. Although MSAs, for example, paclitaxel, have served clinical treatment for years 4, 29, their defects such as poor water solubility, photosensitivity, and drug-resistance development 1, 4, 29 pronounce that novel chemotherapy regimens are imperiously demanded. As a response, we selected ID09, a colchicine derivative with ameliorating effects 13, as our study object and devoted ourselves to estimating the MDA anti-tumor potency in OSCC from various angles. We contrived to substantiate that ID09 suppressed OSCC malignant biological behavior, including proliferation, EMT process, and migration, in a dose-dependent manner. In addition, explorations of both apoptosis fluctuation as well as malignant biological behaviors associated with variations in Mcl-1 and the role Ras-Erk pathway played during this process were also explored. One of the landmarks to malignant biological properties is proliferation, which can shield inherent regulation 30. The images of tumor xenograft, analysis of *in vivo* tumor volume and weight, as well as clone formation assay and soft agar colony formation assay performed *in vitro* pointed to the same conclusion: ID09 incubation restrained tumor growth and cell proliferation. During proliferation, G1/S and G2/M transformations are two essential checkpoints 26, 31, which are enormously active and can be handily obstructed by environmental stimulants, leading to cell cycle arrest and even death 27. In the case of MDAs, the classic concept alleges that the principal mechanism can be interpreted as aberrant spindle formation 6 since microtubules, their major components, are exposed to destabilization 4, 32. As a result, the cell cycle is arrested in the G2/M phase 33. Different from the foretime, we focused anti-proliferation mechanism on PCNA, a ring-shaped homotrimer encircling the DNA 34. Western blot and IHC staining certified that the expression of PCNA and Ki-67 were down regulated by ID09 treatments both *in vivo* and *in vitro*. Congruously, revealing by flow cytometry, the proportions of SCC-15 and Cal-27 cells arrested in both S phase and G2/M phase were distensible. This result demonstrated that ID09 treatment restrained cell proliferation and arrested cell cycle via both PCNA variation and spindle abnormity respectively.

Another malignant biological property of OSCC is EMT, a phenomenon indicating that cancer cells shed epithelial characteristics and translated into a mesenchymal and invasive phenotype 35, 36; also, microtubules contributed to the formation of the cytoskeleton, which had a significant influence on EMT 37. Several molecular markers are partially employed to judge the EMT progress 38. During metastasis, cancer cells can employ the EMT process to augment migration capabilities 39, which are paramount causes of cancer metastasis and spread 40. Relationships exist between cellular morphology and cellular cytoskeleton containing microtubules as a crucial component 39, 41. Meanwhile, evidence suggests that the EMT process can also receives the positive adjustments from Mcl-1 42, 43. Variations tendencies of EMT biomarkers extracted from both cells and tumor tissues detected by qRT-PCR and Western blot demonstrated the reversal of the EMT process due to ID09 treatment. As a consequence, the migration capabilities were also degraded. All of these changes could be ascribed to the disturbance in microtubules, the purpose ID09 designed for.

Dedicating to homeostasis 15, cell apoptosis can be triggered directly by internal and/or external stimulus 14 or indirectly by any anomaly in biological activities, ranging from proliferation, EMT progress, to migration 44-47, all of which were generated by ID09. The kernel of apoptosis is the caspase cascade, involving the cell surface death receptor (caspase-8) pathway and the mitochondria-initiated (caspase-9) pathway 14. Researches demonstrated that the mitochondria-initiated pathway was the major apoptosis approach induced by MDAs, starting with mitochondrial rupture and a drop in membrane potential 48. On the contrary, withstanding the caspase cascade, Bcl-2 subfamily proteins, including Mcl-1, evolved to restrain the release of cytochrome c from mitochondria for the anti-apoptotic effect 17, 18. Our study showed the activation of caspase cascade via the mitochondria-initiated pathway both *in vivo* and *in vitro* after ID09 incubation, implying apoptosis in cells and tumor tissues. Flow cytometry results substantiated it practically. Furthermore, the expression of Mcl-1, which is customarily neglected in studies on MDAs, was examined as an initiation factor of caspase activation. The aforementioned results showed that ID09 stimulated caspase-dependent apoptosis via Mcl-1 down regulation.

A correlation exists between the Ras pathway and apoptosis, proliferation, migration and invasion 22, 49. Meanwhile, a recent study reported that the phosphorylation of Erk1/2 had a significant impact on the expression level of Mcl-1 23, 50. However, whether the pathway mediates apoptosis through regulating Mcl-1 is still a mystery. On the one hand, the gain of function assay illustrates that the reactivation of Ras-Erk pathway suppressed by ID09 results in the retrieve of proliferation, migration and invasion abilities in OSCC cells. Dramatically consistent with the results of apoptosis, our study was novel in demonstrating that ID09 inhibited the activation of the Ras-Erk pathway, which had an impact on OSCC, and identifying the role of the Ras-Erk pathway in Mcl-1 variation. Furthermore, the Erk1/2 phosphorylation agonist Ro 67-7476 was applied to identify the correlation between ID09, Ras-Erk pathway, Mcl-1, and mitochondria-initiated apoptosis from the opposite. What emerged was that the ID09 effects on Erk dephosphorylation, Mcl-1 down regulation, and apoptosis acceleration were obviously counteracted by Ro 67-7476. This phenomenon initially symbolized the decisive status of the Ras-Erk pathway occupied in the working mechanism of MDAs, which was never reported earlier.

It is self-evident that the relationship between tubulin inhibitor-induced apoptosis and Mcl-1 alternation discussed in present study possess initiative. However, several researches reported close correlations between Mcl-1 and other biological abnormalities beyond apoptosis 17, 20. Hence, further exploration is required to uncover the linkage lying in Mcl-1 and other malignant biological behaviors.

**Figure 9:**
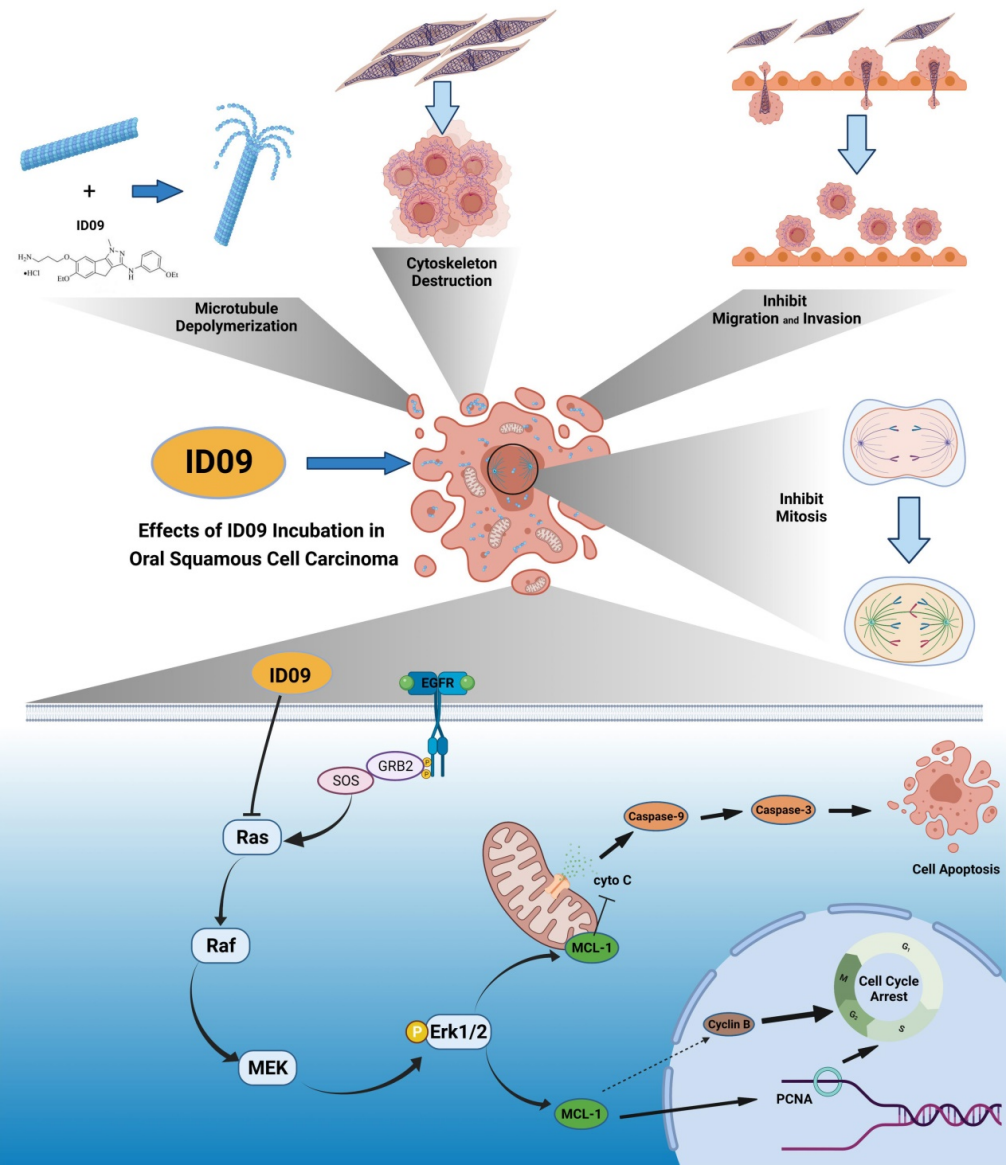
Anti-cancer effects of ID09 against various kinds of carcinomatous properties in OSCC and potential molecular mechanism. Binding to CBS, ID09 can fleetly catalyze microtubule depolymerization and cytoskeleton destruction, resulting in invasion and migration attenuation, EMT process reversion, and mitosis abnormality. In addition, due to deactivation of Ras-Erk pathway, the expression level of Mcl-1 is mitigated by ID09 treatment, resulting in triggering of caspase cascade and mitochondria-initiated apoptosis.

Generally, this study primarily demonstrated the pivotal role of Mcl-1 and the regulatory effect of the Ras-Erk pathway in ID09-induced OSCC cell apoptosis. ID09 restrained the proliferation, migration, and EMT of OSCC cells, and induced mitochondria-initiated apoptosis. Noticeably, since the deactivation of the Ras-Erk pathway, the expression level of Mcl-1 was down regulated, resulting in ID09-induced apoptosis (Fig. 9), which could be offset by the Erk1/2 agonist Ro 67-7476. In summary, this study clarified the approaches and mechanism of ID09 restraining OSCC, thus guiding the development of OSCC chemotherapy.

## Author Contributions

Author Contributions: Conceptualization and design, Q. F. and P. Y.; data administration, Q. F. and P. Y.; formal analysis, H. W. , C. L., T.H. and Z. L.; capital acquisition, M. L. ; investigation, C. L. and H. W.; methodology counsel, M. L. ,T.H. and Z. L.; project management, M. L. ; resources, M. L.; software operation, Q. F. and P. Y.; supervision, M. L.; validation, Q. F. and M. L. ; visualization C. L. and H. W. ; writing - original draft Q. F., P.Y. and H. W.; writing - review & editing, C. L. , Z. L. T.H. and M. L. All authors have read and agreed to the published version of the manuscript.

## Supplementary Material

Supplementary figure.

## Abbreviations

OSCC: oral squamous cell carcinoma
EMT: epithelial-mesenchymal transition
CCK-8: Cell Counting Kit-8
qRT-PCR: quantitative reverse transcription polymerase chain reaction
Mcl-1: Myeloid-cell leukemia 1
Bcl: B-cell lymphoma
Erk: extracellular regulated protein kinases
Raf: rapidly accelerated fibrosarcoma
PTX: paclitaxel
MDA: microtubule-destabilizing agent
MSA: microtubule stabilizing agents
CBS: colchicine-binding site
PCNA: proliferating cell nuclear antigen
MMP: matrix metalloproteinase
PARP: poly ADP-ribose polymerase
DMEM: Dulbecco's modified Eagle's medium
PBS: phosphate-buffered saline
FBS: fetal bovine serum
PI: propidium iodide
BSA: bovine serum albumin
HE: htoxylin eosin
IHC: Immunohistochemistry
MMP: mitochondrial membrane potential
