# Supplementary material for: ID09, A Newly-Designed Tubulin Inhibitor, Regulating the Proliferation, Migration, EMT Process and Apoptosis of Oral Squamous Cell Carcinoma
Source: Int J Biol Sci. 2022 Jan 1;18(2):473–90. doi: 10.7150/ijbs.65824 (PMC8741845; doi:10.7150/ijbs.65824)
Supplement: Supplementary file 1 — Supplementary figure. [file ijbsv18p0473s1.pdf]

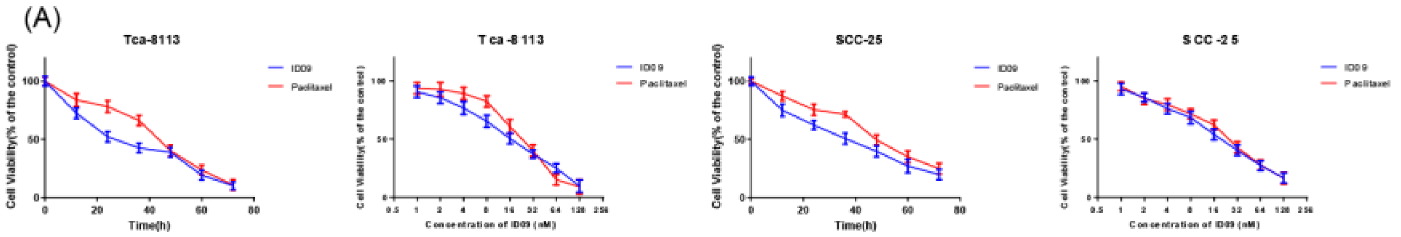

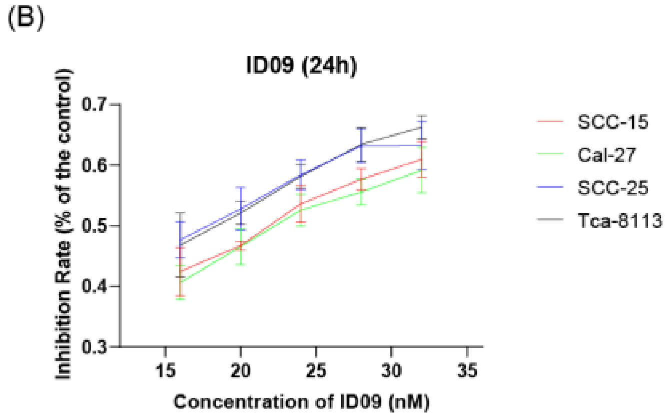

(C)

Table 1. Linear regression analysis of IC_{50}

Cell Line	Regression Equation	R Value	IC_{50}
SCC-15	Y = 0.01202*X + 0.2345	0.9848	22.09 nM
Cal-27	Y = 0.01154*X + 0.2322	0.9776	23.21 nM
Tca8113	Y = 0.01257*X + 0.2719	0.9869	18.15 nM
SCC-25	Y = 0.01042*X + 0.3207	0.9736	17.21 nM

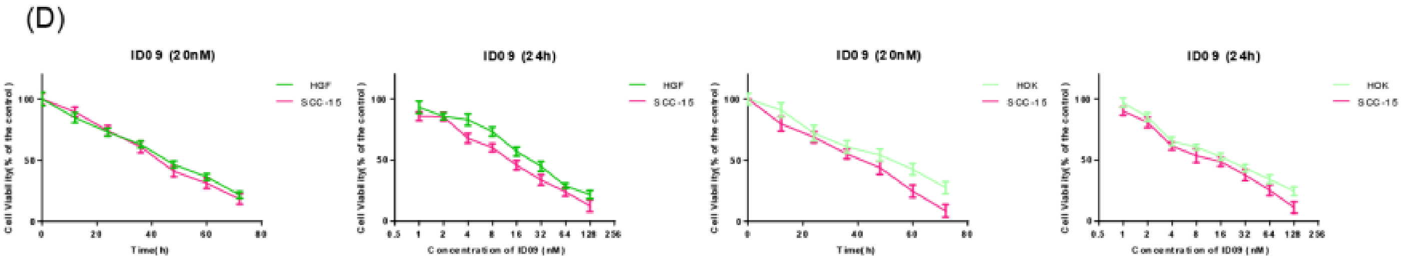

Supplementary data 1. (A) Cell viabilities of two other OSCC cell lines Tca8113 and SCC-25 influenced by ID09 in dose and time dependent manner were detected by CCK8 assay. (B and C) IC_{50} of ID09 in four kinds of OSCC cell lines including SCC-15, Cal-27, Tca8113 and SCC-25 were measured by CCK8 and calculated via linear-regression analysis. (D) CCK8 was selected to compare cell viability between normal non-cancerous oral cells (HOK and HGF) and SCC-15 cell line incubated with ID09 in dose and time dependent manner. The columns represent the means and error bars represent standard deviations. (NS, non-significant difference; */#P < 0.05; **/##P < 0.01; ***/### P < 0.001 versus the indicated group).
